# Supplementary material for: In Silico Screening of Drugs That Target Different Forms of E Protein for Potential Treatment of COVID-19
Source: Pharmaceuticals (Basel). 2023 Feb 14;16(2):296. doi: 10.3390/ph16020296 (PMC9958997; doi:10.3390/ph16020296)
Supplement: Supplementary file 1 [file pharmaceuticals-16-00296-s001.zip › TableS1.pdf]

Table S1. Molecular docking studies drugs on the E-homopentamer (Ion Channel): The best affinities calculated from molecular docking analysis (on the Ion Channel form) are reported in this table.

**Molecular docking studies drugs on the E-homopentamer (Ion Channel).**		
Drug	Affinity (kcal/mol)	Amino acids involved
Lumacaftor	-10.9	Phe26E, Phe26A, Phe23E, Val29A, Val25A, Ala22E, Leu19E, Ala22C, Ala22B, Ala22D, Phe23C, Phe26D, Phe26C, Phe26B, Val25D, Leu19C, Ala22A
Xaliproden	-10.9	Phe26D, Phe26A, Leu27E, Thr30E, Val29A, Phe23B, Val29C, Phe26C, Phe26B, Phe26E, Phe23E
Nilotinib	-10.8	Leu28D, Leu27C, Val25D, Leu19A, Phe23A, Phe26B, Phe26A, Phe26D, THr30C, Phe26E, Val29C, Ala22B, Ala22D, Phe26C, Ala22C, Leu19C, Ala22A, Val29D, Phe23C
Avodart	-10.6	Leu19D, Leu18D, Leu19C, Leu18C, Ser16B, Leu21C, Phe26A, Leu19B, Leu18B, Phe26E, Phe23E, Val25A, Leu19E, Ala22A, Ala22C
Naldemedine	-10.5	Ala22A, Leu19E, Phe26A, Leu18D, Leu19C, Phe26E, Phe23E, Ala22E, Val25A, Val29A, Phe26D, Val25D, Phe23C, Ala22C, Phe26C, Leu19D, Ala22D, Phe26A
Ebastine	-10.3	Phe23C, Thr30C, Val29C, Thr30E, Leu27E, Val29A, Phe26D, Phe26C, Phe26E, Phe26A, Phe23E, Val29D, Val25D, Val25A.
Pranlukast	-10.3	Leu18C, Val25A, Leu19E, Thr30E, Leu27E, Asn15B, Leu18B, Leu19B, Val29A, Ala22C, Leu21C, Leu18A, Ala22A, Ala22E, Phe26A, Phe23E, Phe26E.
Zafirlukast	-10.2	Val29C, Phe26C, Phe26D, Val29D, Val29A, Val25D, Phe26A, Phe26B, Phe26E, Phe23E, Thr30C, Thr30B, Ile33C, Phe23C, Val29E, Thr30E
Irinotecan	-10.1	Ala22D, Phe26E, Leu19C, Leu18C, Leu19B, Leu28D, Thr30C, Leu18B, Leu19B, Leu18C, Phe23C, Val25D, Leu27C, Val29D, Ala22C, Phe26C, Phe26D
Flunarizine	-10.1	Leu19C, Leu19B, Phe23B, Phe26B, Phe23B, Phe26D, Leu19E, Ala22E, Ala22B, Ala22C, Ala22D, Val29D, Val25A, Phe26C, Phe23E, Phe26E, Phe26A,
Rolapitant	-10.1	Phe26E, Ala22D, Phe26D, Phe26C, Ala22E, Val25A, Leu19D, Ala22C, Phe23C, Leu19C, Val25D, Ala22A, Leu18B, Leu19B, Ala22B, Leu19A, Leu19E, Phe26A y Phe23E
Conivaptan	-10.1	Asn15E, Phe26A, Phe26E, Leu18D, Leu19c, Leu19B, Phe26C, Phe26D, Ala22A, Leu21A, Leu19E, Leu18A, Ala22E, Ala22D, Leu19D, Leu18E, Ala22C
Mepron	-10	Leu18A, Leu19E, Thr30E, Leu18D, Leu19D, Phe23E, Leu18E, Phe26E, Ala22A, Ala22E, Phe26A, Val25A y Val29A
Apc	-10	Phe23E, Ala22E, Phe26E, Val25A, Val29A, Thr30C, Leu28D, Phe26A, Phe26D, Phe26C, Leu27C, Val25D, Val29D, Phe23C.
Saquinavir	-9.9	Phe26B, Ala22B, Leu19A, Phe26C, Phe26D, Phe23C, Ala22D, Leu18D, Leu19C, Phe26A, Leu18E, Leu19D, Leu18B, Leu19E, Val25A, Phe23E, Leu18C, Asn15B, Leu21C, Ala22C, Ala22A, Leu18A, Ala22E, Leu19B, Phe26E
