# Supplementary material for: In Silico Screening of Drugs That Target Different Forms of E Protein for Potential Treatment of COVID-19
Source: Pharmaceuticals (Basel). 2023 Feb 14;16(2):296. doi: 10.3390/ph16020296 (PMC9958997; doi:10.3390/ph16020296)
Supplement: Supplementary file 1 [file pharmaceuticals-16-00296-s001.zip › TableS2.pdf]

Table S2. Molecular docking studies drugs on the E-homopentamer (N-terminal): The best affinities calculated from molecular docking analysis (on the E-homopentamer (N terminal)) are reported in this table

Molecular docking studies drugs on the E-homopentamer (N-terminal).		
Drug	Affinity (kcal/mol)	Amino acids
Alectinib	-9.6	Asn15E, Val14E, Asn15D, Thr11D, Asn15, Glu7B, Thr11A, Thr11B, Glu7B, Gly10B, Leu12A, Ser6B, Val5B, Glu7A, Glu8A, Ser3B
Saquinavir	-9.3	Ser3D, Tyr2D, Val5D, Glu7C, Met1D, Gly10C, Ser6C, Ser6B, Glu8B, Gly10B, Val5B, Glu8A, Glu7B, Thr11A, Thr11B, Asn15A, Thr11E, Gly10E, Val14E, Thr11D, Glu8D, Glu7D
Amaryl	-9.2	Glu7A, Glu8A, Val5B, Leu12A, Gly10B, Thr11A, Ser6B, Glu7B, Thr11C, Val14C, Tyr2C, Val5C, Ile13C, Gly10C, Glu8B, Met1C, Ser6C
Ponatinib	-9.2	Glu7D, Ser6D, Val5D, Gly10C, Val14D, Asn15D, Thr11D, Thr11C, Asn15A, Thr11A, Ser6B, Gly10B, Leu12A, Val5B, Glu8A, Glu7A, Ser3B, Glu7B, Thr11B
Lifitegrast	-9.2	Met1C, Ser3B, Ser6B, Glu8A, Glu7B, Gly10B, Thr11A, Met1A, Thr11E, Glu7D, Val5E, Glu8D, Gly10E, Val14E, Met1E, Thr11D, Asn15A, Thr11B, Glu7A
Accolate	-9.1	Thr11E, Glu7D, Met1A, Thr11A, Glu7B, Thr11D, Asn15D, Met1D, Glu7C, Val5D, Glu8C, Gly10C, Thr11C, Ser6D, Val14D, Asn15A, Gly10E, Glu10E, Glu8D
Lumacaftor	-9.1	Leu12B, Glu7B, Glu8B, Met1D, Glu7D, Val5D, Ser6D, Gly10D, Thr11D, Val14D, Gly10D, Thr11C, Val14C, Ser6C, Ile13C, Val5C, Tyr2C
Vinblastine sulfate	-9.1	Glu7E, Met1E, Glu8D, Glu7D, Gly10C, Thr11C, Ser6D, Val5D, Val14D, Gly10D, Thr11D, Thr11B, Glu7B, Met1D, Met1C, Ser6C, Glu8B, Thr11E, Thr11A, Glu7A, Met1A, Glu8E

.
