# Supplementary material for: In Silico Screening of Drugs That Target Different Forms of E Protein for Potential Treatment of COVID-19
Source: Pharmaceuticals (Basel). 2023 Feb 14;16(2):296. doi: 10.3390/ph16020296 (PMC9958997; doi:10.3390/ph16020296)
Supplement: Supplementary file 1 [file pharmaceuticals-16-00296-s001.zip › TableS3.pdf]

Table S3. Docking molecular from compounds on the E-monomer (C-terminal solvent exposed): The best affinities calculated from molecular docking analysis on the E-monomer (C-terminal, solvent exposed).

**Docking molecular from compounds on the E-monomer (C-terminal solvent exposed).**		
Drug	Affinity (kcal/mol)	Amino acids
Irinotecan	-8.0	Leu51, Lys53, Phe56, Tyr57, Ser60, Asn64, Lys63, Arg69, Val75, Asp72, Ser67, Ser68, Val70
Saquinavir	-7.9	Val70, Asp72, Ser67, Arg69, Ser68, Val75, Leu74, Tyr57, Lys53, Phe56, Leu51, Ser60, Lys63, Asn64, Tyr59
Avodart	-7.7	Leu74, Pro71, Val70, Asp72, Ser68, Ser67, Arg69, Lys63, Val75, Asn64, Ser60, Tyr59, Phe56
Novobiocin sodium	-7.7	Val52, Ser55, Tyr59, Ser60, Lys63, Asn64, Phe56, Ser67, Ser68, Val70, Asp72, Arg69, Leu74, Val75
Lapatinib	-7.6	Lys63, Leu74, Asp72, Val75, Ser67, Ser68, Val70, Arg69, Phe56, Ty57, leu51, Lys53, Ser60, Asn64, Tyr59
Tetracycline hydrochloride	-7.5	Val70, Ser67, Asp72, Arg69, Val75, Ser68, Asn64, Leu74
Cepharanthine	-7.5	Ser60, Asn64, Leu74, Lys63, Val75, Ser67, Arg69, Asp72, Val70, Leu74
Pranlukast	-7.5	Ser55, Tyr59, Phe56, Lys63, Ser67, Arg69, Asp72, Ser68, Val70, Val75, Asn64, Ser60
Naldemidine	-7.4	Lys63, Leu74, Asp72, Asn64, Ser67, Ser68, Ser60, Arg69, Tyr59, Val75
Ixabepilone	-7.3	Asn64, Arg69, Ser67, Ser68, Lys63, Asp72, Leu73, Pro71, Val70, Leu74, Val75
Imatinib	-7.3	Tyr59, Ser60, Asn64, Arg69, Ser68, Ser67, Lys63, Pro71, Asp72, Val70, Leu73
