# Supplementary material for: In Silico Screening of Drugs That Target Different Forms of E Protein for Potential Treatment of COVID-19
Source: Pharmaceuticals (Basel). 2023 Feb 14;16(2):296. doi: 10.3390/ph16020296 (PMC9958997; doi:10.3390/ph16020296)
Supplement: Supplementary file 1 [file pharmaceuticals-16-00296-s001.zip › TableS4.pdf]

Table S4. Docking molecular from compounds on the E-monomer (C-Terminal Harpin): The best affinities calculated from molecular docking analysis on the E monomer (C-terminal Hairpin).

**Docking molecular from compounds on the E-monomer (C-Terminal Harpin).**		
Drug	Affinity (kcal/mol)	Amino acids
Irinotecan	-8.2	Phe20, Phe23, Leu27, Leu73, Leu74, Val47, Val58, Ile46, Val49, Leu51, Tyr57, Ser55, Tyr59
Nilotinib	-8.2	Ser55, Tyr57, Tyr59, Leu74, Val75, Leu51, Val58, Val62, Arg69, Pro71, Val70
Avodart	-8.1	Val47, Leu27, Ile46, Leu51, Val70, Try57, Val58, Val49, Val75, Leu74, Pro71
Fexofenadine	-8.0	Val58, Val62, Val70, Try59, Val75, Tyr57, Ser55, Leu51, Leu65, Arg61, Ser67, Arg69, Pro71, Asp72
Ternadin	-8.0	Tyr57, Ser55, Val58, Leu51, Tyr59, Val75, Val62, Leu65, Ser67, Arg61, Ser68, Arg69, Val70, Asp72
Lumacaftor	-7.9	Ser55, Leu51, Val62, Ser68, Arg69, Tyr57, Pro71, Val75, Leu74, Val70, Val58
Amaryl	-7.8	Ser55, Tyr57, Val58, Val70, Pro71, Leu74, Val75, Val49, Leu27, Val47, Ile46, Ser50, Leu51
Saquinavir	-7.7	Val70, Arg61, Pro71, Tyr57, Leu74, Leu51, Phe56, Val52, Ser55, Tyr59, Val75, Val58
Naldemidine	-7.7	Lys63, Leu65, Arg69, Ser67, Ser68, Val70, Arg61, Leu74, Val62, Try59, Val58, Val75
