# Supplementary figures and images for: In Silico Screening of Drugs That Target Different Forms of E Protein for Potential Treatment of COVID-19
Source: Pharmaceuticals (Basel). 2023 Feb 14;16(2):296. doi: 10.3390/ph16020296 (PMC9958997; doi:10.3390/ph16020296)

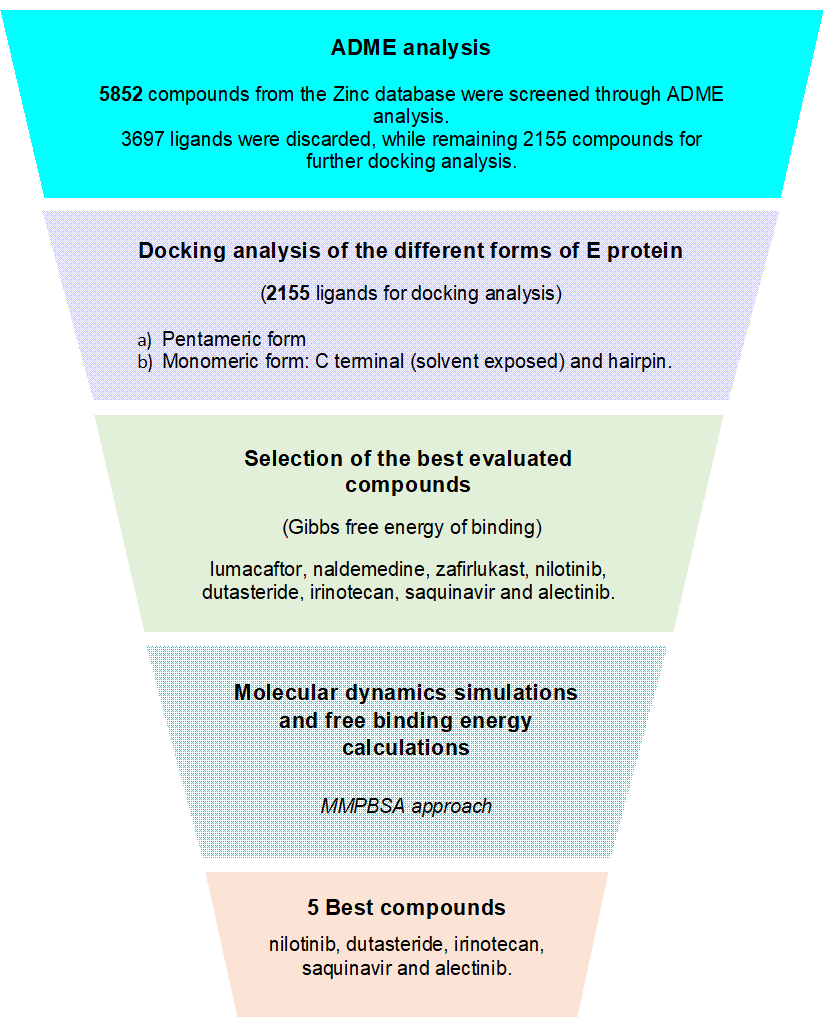

Supplement: Supplementary file 1 [file pharmaceuticals-16-00296-s001.zip › FigureS1.png]

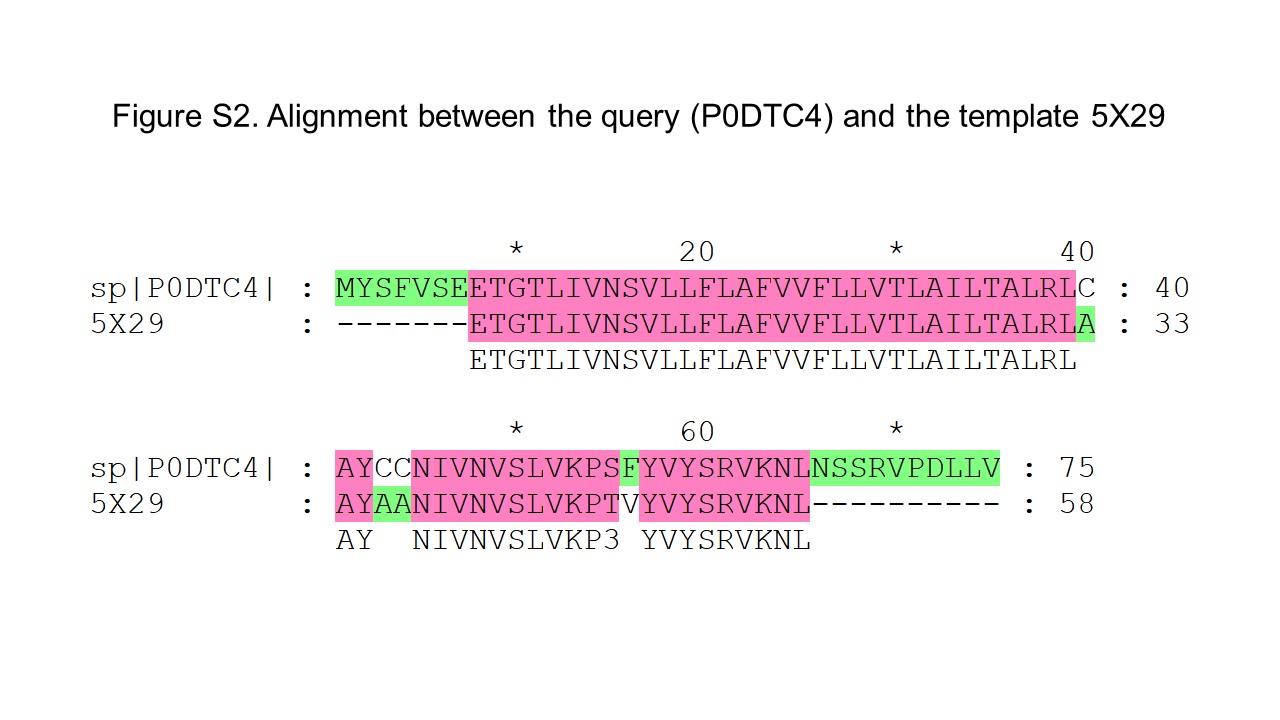

Supplement: Supplementary file 1 [file pharmaceuticals-16-00296-s001.zip › FigureS2.jpg]
