# Supplementary material for: In Silico Screening of Drugs That Target Different Forms of E Protein for Potential Treatment of COVID-19
Source: Pharmaceuticals (Basel). 2023 Feb 14;16(2):296. doi: 10.3390/ph16020296 (PMC9958997; doi:10.3390/ph16020296)
Supplement: Supplementary file 1 [file TableS5.pdf › TableS5.pdf]

Table S5: Pharmacokinetic properties. The ADMET properties of the selected drugs are shown.

Pharmacokinetic properties								
Molecule	Lumacaftor	Irinotecant	Alectinib	Saquinavir	Avodart	Nilotinib	Naldemedine	Zafirlukast
Molecule properties								
MW	452.41	586.68	482.62	670.84	528.53	529.52	570.64	575.68
Lipinski #violations	0	1	0	2	2	1	1	1
PAINS #alerts	0	0	0	0	0	0	0	0
Brenk #alerts	0	0	0	0	0	0	0	2
Synthetic Accessibility	3.28	5.59	3.92	5.94	5.36	3.81	6.29	4.36
Absorption								
Consensus LogP	4.06	3.73	4.33	2.87	5.71	4.63	2.98	5.35
Silicos-IT LogSw	-7.81	-7.28	-8.4	-8.54	-7.47	-10.86	-7.07	-8.87
GI absorption	High	High	High	Low	Low	Low	Low	Low
Distribution								
BBB permeant	No	No	Yes	No	No	No	No	No
Pgp substrate	Yes	Yes	Yes	Yes	Yes	No	No	No
Metabolism								
CYP1A2 inhibitor	Yes	No	No	No	No	No	No	No
CYP2C19 inhibitor	Yes	No	Yes	No	No	Yes	No	No
CYP2C9 inhibitor	Yes	Yes	Yes	No	No	Yes	No	Yes
CYP2D6 inhibitor	Yes	Yes	No	No	No	Yes	Yes	No
CYP3A4 inhibitor	Yes	Yes	No	Yes	Yes	Yes	Yes	No
Excretion								
Bioavailability Score	0.56	0.55	0.55	0.17	0.17	0.55	0.55	0.55
Toxicity								
Mutagenic	none	none	none	none	none	none	none	none
Tumorigenic	none	none	none	none	none	none	none	none
Reproductive Effective	none	none	none	none	none	none	none	none
Irritant	none	none	none	none	none	none	none	none
